# A Unique Interactive Nanostructure Knitting based Passive Sampler Adsorbent for Monitoring of Hg^2+^ in Water

**DOI:** 10.3390/s19153432

**Published:** 2019-08-05

**Authors:** Raghuraj S. Chouhan, Gregor Žitko, Vesna Fajon, Igor Živković, Majda Pavlin, Sabina Berisha, Ivan Jerman, Alenka Vesel, Milena Horvat

**Affiliations:** 1 Institute “Jožef Stefan”, Department of Environmental Sciences, Jamova 39, 1000 Ljubljana, Slovenia; 2 National Institute of Chemistry, Hajdrihova 19, 1000 Ljubljana, Slovenia; 3 Jožef Stefan International Postgraduate School (IPS), Jamova 39, 1000 Ljubljana, Slovenia; 4 Institute “Jožef Stefan”, Department of Surface Engineering, Jamova 39, 1000 Ljubljana, Slovenia

**Keywords:** nanosheets, adsorption, passive sampler, mercury, analysis

## Abstract

This work reports the development of ultralight interwoven ultrathin graphitic carbon nitride (g-CN) nanosheets for use as a potential adsorbent in a passive sampler (PAS) designed to bind Hg^2+^ ions. The g-CN nanosheets were prepared from bulk g-CN synthesised via a modified high-temperature short-time (HTST) polycondensation process. The crystal structure, surface functional groups, and morphology of the g-CN nanosheets were characterised using a battery of instruments. The results confirmed that the as-synthesized product is composed of few-layered nanosheets. The adsorption efficiency of g-CN for binding Hg^2+^ (100 ng mL^−1^) in sea, river, rain, and Milli-Q quality water was 89%, 93%, 97%, and 100%, respectively, at natural pH. Interference studies found that the cations tested (Co^2+^, Ca^2+^, Zn^2+^, Fe^2+^, Mn^2+^, Ni^2+^, Bi^3+^, Na^+^, and K^+^) had no significant effect on the adsorption efficiency of Hg^2+^. Different parameters were optimised to improve the performance of g-CN such as pH, contact time, and amount of adsorbent. Optimum conditions were pH 7, 120 min incubation time and 10 mg of nanosheets. The yield of nanosheets was 72.5%, which is higher compared to other polycondensation processes using different monomers. The g-CN sheets could also be regenerated up to eight times with only a 20% loss in binding efficiency. Overall, nano-knitted g-CN is a promising low-cost green adsorbent for use in passive samplers or as a transducing material in sensor applications.

## 1. Introduction

Mercury remains one of the most hazardous pollutants causing severe health problems in humans and animals [1]. Furthermore, mercury is harmful at trace concentrations and in its organic form can bioaccumulate and biomagnify up the food chain as highly toxic methylmercury (MeHg) [1]. Despite the work of the Minamata Convention, many people remain exposed to potentially hazardous levels of mercury. For example, mercury that is used to extract the gold from the ore in artisanal gold mining remains in the tailings dam after the washing process, where it settles out and leaches into the groundwater [2]. The communities (often poor and remote) living near these gold mines are exposed to the mercury in the water and soil and to the MeHg in their diet, which can have serious health implications, especially for children. The long-term monitoring of Hg in these often isolated and remote locations creates a need for passive samplers (PAS) made from cheap, robust, and selective adsorbent materials.

The literature reveals several types of adsorbents that have been investigated for monitoring or remedial applications regarding metal ions in water. These include carbon nanotubes [3,4,5], clay minerals [6], zeolites [7], natural and modified bentonite [8], graphene [9,10], and functionalized adsorbents [11,12,13]. The latter have been shown to successfully remove cesium [14,15] palladium [16], cobalt [17], mercury [18], dysprosium and lutetium [19], and copper [20] from wastewater.

The disadvantage of these materials is their complicated conjugation chemistries and poor reusability, which limits their application. These materials, although effective adsorbents, either require a significant number of sample preparation steps, large volumes, or are cost-prohibitive. The search for an ideal adsorbent material, which is cheap, inexpensive, earth-abundant, green, and is capable of selectively trapping mercury, remains a challenge.

One possible material that could meet this challenge is graphitic carbon nitride g-CN. Graphitic carbon nitride is the most stable allotrope of carbon nitride and has a high bandgap energy (2.76 eV), high porosity, a high number of surface active sites, is earth-abundant, has minimal surface defects and is non-toxic and metal-free [21]. Moreover, nanosheets derived by the delamination of 2D layered compounds are regarded as a novel class of nanostructured materials owing to their unique structural attributes and ultimate two-dimensional anisotropy with extremely small (nano-scale) thicknesses. These nanosheets also have unique physicochemical properties due to the quantum confinement effect [21]. For instance, they possess exceptional mechanical, electronic, thermal, optical properties compared to bulk nanomaterials [21,22]. Similar to graphite, g-CN also has a layered structure with weak van der Waals interaction between the adjacent C-N layers. Also, the g-CN planes, constructed via highly ordered tri-s-triazine units, contain many ideal coordination sites, where metal ions can interact with the lone-pair electrons of nitrogen [23,24,25]. The single or few-layer structures made up of thin layers with short inter-layer distances result in fast carrier transport of metal ions [26,27,28]. Also, the triazine units, assembled on the thin lamellar structure, create a unique active nanostructure that has an ultralight “knitted” structure, with a large surface area containing functional coordination bonds ideal for capturing Hg^2+^. Importantly, g-CN materials are environmentally friendly, abundant and inexpensive to produce [29]. So far, the practical application of g-CN (bulk and nanosheets) has been limited to photocatalytic water splitting, photodegradation of dyes, bio-imaging, photocatalyst and fluorescence applications [30,31]. It also has potential as an efficient adsorbent for use in PAS and as a transducer material in sensor applications. In this study, we propose an efficient adsorbent material based on ultrathin g-CN stratified interwoven “knitted” nanosheets (prepared via a novel exfoliation method) for adsorbing Hg^2+^.

## 2. Material and Methods

### 2.1. Reagents

A SnCl_2_ (5% *w*/*v*) solution was prepared from SnCl_2_·2 H_2_O (analytical grade, Merck, Darmstadt, Germany) in Milli-Q water containing 3 M H_2_SO_4_ (Merck, Darmstadt, Germany). A H_2_SO_4_ acid solution (0.1 M) was prepared from H_2_SO_4_ (Merck, Darmstadt, Germany) in Milli-Q water; a KMnO_4_ solution (0.5% *w*/*v*) was prepared from KMnO_4_ (analytical grade, Merck, Darmstadt, Germany) in Milli-Q water; and a NaOH solution (10 M) was prepared from NaOH (extra pure, Merck, Darmstadt, Germany) in Milli-Q water. The dicyandiamide monomer (99%) was purchased from Sigma-Aldrich. All other reagents are of analytical grade and used as supplied.

### 2.2. Sample Preparation

A standard stock solution of Hg^2+^ (10 mg mL^−1^) was prepared by dissolving an appropriate amount of HgCl_2_ (Merck, Darmstadt, Germany) in 0.1 M HCl in isopropanol. Working solutions (10, 100, and 1000 ng mL^−1^) were prepared by appropriate dilution of the stock solutions with Milli-Q water. Working and stock solutions of Hg^2+^ were prepared daily and stored in the dark at 4 °C. All glassware was cleaned by soaking for 12 h in a 5% detergent solution (Micro-90, Thermo Scientific, Strasbourg, France), rinsed with regular tap water followed by Milli-Q water and stored in a sealed container. All reaction vessels were placed in 50% (*v*/*v*) concentrated HNO_3_ solution and heated at 55 °C for two days. After rinsing with Milli-Q water, the glassware was soaked in 10% (*v*/*v*) HCl for 24 h at room temperature, rinsed with Milli-Q water, filled with 1% HCl, and stored in polyethylene plastic bags.

### 2.3. Synthesis of g-CN Nanosheets

Nanosheets were prepared from the bulk g-CN powder, which was synthesised from the monomer according to [27] and [28]. In brief, 5 g of dicyandiamide (99%, Sigma Aldrich, St. Louis, MO, USA) was dried in an inert atmosphere for 1 h, placed in a closed container and transferred to a preheated furnace oven at 600 °C for 2 h. The as-obtained product was then left to cool to room temperature. The resultant pale yellowish agglomerate was milled to a fine powder with a mortar and pestle. In this paper, a novel method of thermal oxidation was used to produce the nanosheets. This approach avoids the use of harsh chemicals [32] and is high-temperature short-time (HTST). Also, this method does not result in any unexfoliated residue. Next, 3 g of the bulk g-CN was placed in a ceramic container, covered with aluminium foil and heated to 550 °C for 2 h at 5 °C/min. The result was a light yellow powder consisting of g-CN nanosheets. The yield of the final product was 72.5%, which is higher than that reported in the literature [32].

### 2.4. Characterization

X-ray diffraction (XRD) patterns were recorded using a high-resolution X-ray powder diffractometer (PANalytical X’Pert PRO) at a wavelength of CuKα1 = 1.5406 Å. Nanosheet structures were determined using a Supra 35 VP scanning electron microscope (SEM) and ARSTEM—Atomic resolution Cs corrected scanning transmission electron microscope (Jeol, JEM-ARM200CF, Peabody, MA, USA). Height profile and the graphitic nature of the g-CN nanosheets were examined using a Witec alpha 300 A atomic force microscope (AFM). Fourier transform infrared (FTIR) spectra were obtained using an IFS 66/S spectrometer (Bruker) equipped with an integrating sphere (OPTOSOL) while X-ray photoelectron spectroscopy (XPS) measurements of the nanosheets chemical composition were performed using targeted factor analysis, TFA (Physical Electronics). The pressure in the XPS analysis chamber was approximately 6 × 10^−8^ Pa. The samples were excited over a spot area of 300 mm^2^ using monochromatic AlKα1, 2 radiation at 1486.6 eV. The generated photoelectrons were then detected with a hemispherical analyser positioned at 45° to the surface normal. The energy resolution was approximately 0.5 eV. Survey-scan spectra were acquired with a pass energy of 187.85 eV, while for C1s, individual high-resolution spectra were recorded with a pass energy of 29.35 eV and an energy step of 0.125 eV. All spectra were referenced to the main C1s peak of the carbon atoms, which was assigned a value of 284.8 eV. The spectra were analysed using MultiPak v8.1c software (Ulvac-Phi Inc., Kanagawa, Japan, 2006) from Physical Electronics. The C1s spectra were fitted with asymmetrical, Gauss–Lorentz function. Shirley-type background subtraction was used.

### 2.5. Cold Vapour Atomic Absorption Spectrometry (CVAAS)

#### 2.5.1. Determination of Hg^2+^

After 120 min incubation, the Hg^2+^ solution (100 ng mL^−1^) with added nanosheets was centrifuged at 11,000 rpm for 25 min at 22 °C, and the supernatant transferred to a clear glass vial for analysis. The concentration of Hg^2+^ was determined by CVAAS using a mercury analyser Model Hg-201 (Sanso Seisakusho Co. Ltd., Tokyo, Japan). An aliquot (1 mL) of the sample was transferred to the reaction vessel, where it was reduced using a Tin (II) chloride solution and aerated until equilibrium was reached. Acidic gasses were removed using an acid-gas trap (10% NaOH solution). The mercury vapour was then introduced into the absorption cell via a four-way valve [33,34]. The amount of Hg^2+^ in the aqueous phase was calculated by comparing the peak height of the sample with the corresponding peak height of the Hg^2+^ standard traceable to the NIST 3133 standard.

#### 2.5.2. Determining the Amount of Adsorbed Hg^2+^

**Scheme 1:**
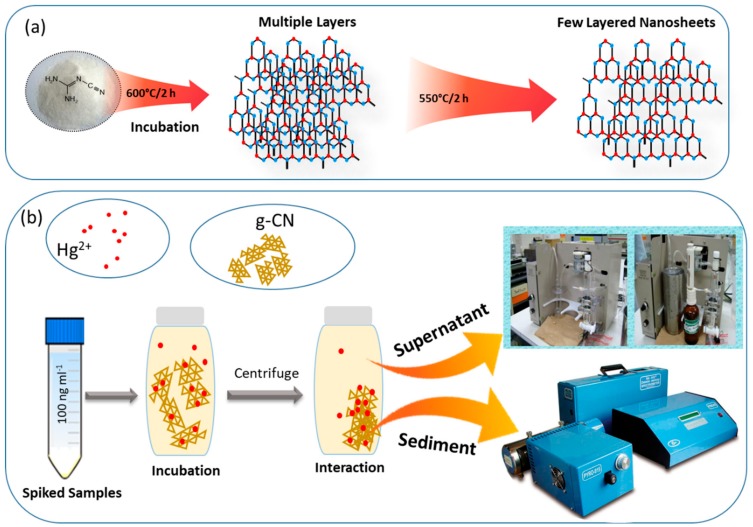
Schematic representation of the present work. (**a**) synthesis of nanosheets using the high-temperature short-time (HTST) polycondensation process, (**b**) overall method used to analyze Hg^2+^.

After centrifugation, the remaining g-CN pellet was dried in a desiccator. The total Hg content was then measured with an RA-915+ Mercury Analyzer fitted with a pyrolysis cell (Lumex-Marketing Ltd., St. Petersburg, Russia). The instrument is based on differential Zeeman atomic absorption spectrometry using high-frequency light polarization modulation [35]. The g-CN sample was placed into a quartz boat, which was inserted into the evaporator and heated to 800 °C. The released Hg was then passed through the catalytic converter, where bound Hg becomes released and detected in the analytical cell. The amount of Hg in the g-CN material was calculated by comparing the peak area of the sample with the corresponding peak area of the standard reference material NIST 3133. To calculate the overall mass balance, the mass of Hg determined in the aqueous phase was summed with the mass of Hg in g-CN material. The sum was then compared to the theoretical mass of Hg added before equilibration. All experiments were performed in triplicate. The overall experimental setup and mode of analysis used in the present work are shown in Scheme 1.

#### 2.5.3. Regeneration

After adsorption, the desorption of Hg^2+^ on g-CN was performed by washing g-CN with a 0.5 M HCl solution followed with 90% ethanol. The finer nanosheets of g-CN were shaken (3 h), centrifuged (12,000 rpm for 25 min), and then washed three times with Milli-Q quality water. The clean sample was then dried in a vacuum oven at 30 °C, after which the g-CN sample was ready to use. This whole procedure of adsorption and desorption was repeated for ten cycles.

## 3. Results and Discussion

### 3.1. Characterisation of the As-synthesized g-CN Nanostructure Material

**Figure 1:**
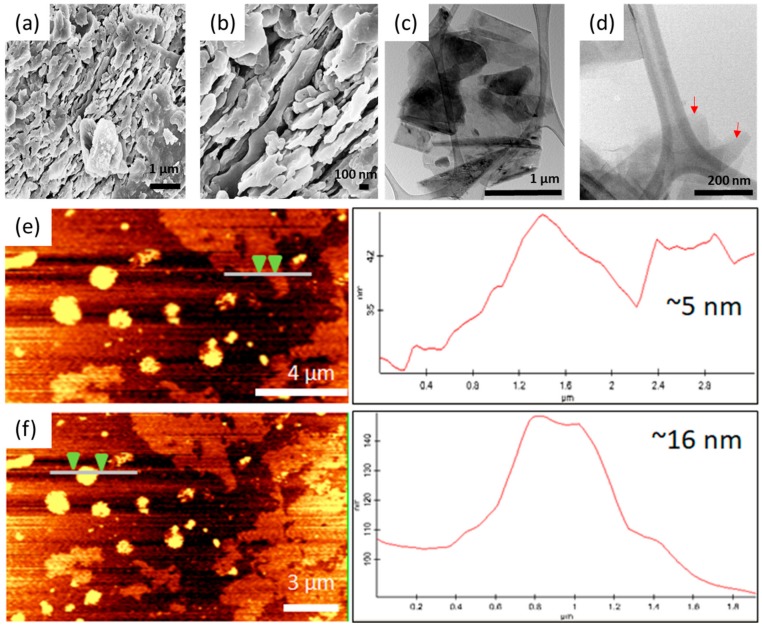
Morphological characteristics of as-synthesized graphitic carbon nitride (g-CN) nanosheets. SEM images of g-CN nanosheets showing few-layered lamellar piled together (**a** and **b**). HR-TEM image of g-CN with low (**c**) and high (**d**) magnification. AFM images of g-CN nanosheets and the corresponding height profiles of different regions (**e** and **f**).

The morphology of the as-synthesized nanosheets was investigated using SEM, TEM, and AFM, which confirmed the existence of a few layered nanosheet structures (Figure 1a–f), and the intactness and visual appearance of the as-synthesized product. Figure 1a shows how the g-CN nanosheets in bulk material are formed from densely packed agglomerated sheets. The sample was then further exfoliated using enhanced polycondensation incubation. In the image are stacked piles of individual sheets with slightly curved edges in a loose lamellar structure (Figure 1b). The observed structure agrees with that reported in the literature [27,36]. TEM images show even finer details of the as-synthesized g-CN nanosheets revealing twisted layers in the centre of the TEM grid (Figure 1c). At higher resolution, the transparent nature of the individual nanosheets becomes apparent (Figure 1d) [37]. From the AFM images (Figure 1e,f) and respective height profiles, the thickness of the g-CN nanosheets is approximately 5 and 16 nm.

**Figure 2:**
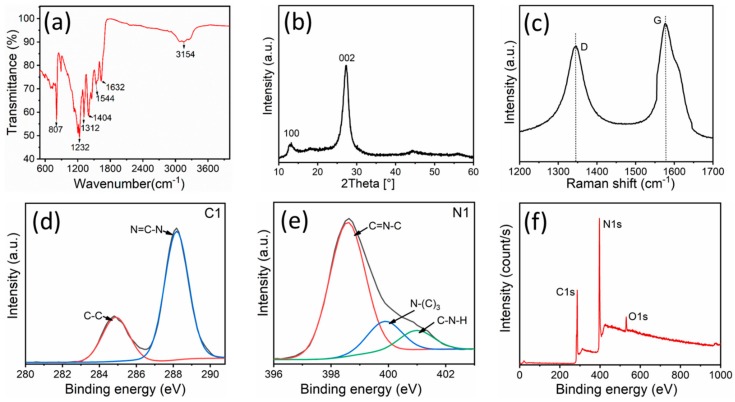
Structural characteristics of as-synthesized nanosheets. (**a**) FTIR spectra of g-CN nanosheets at the frequency range of 500–4000 cm^−1^, (**b**) XRD spectra of g-CN nanosheets, (**c**) Raman signature profile of the nanosheets with distinct D and G bands, (**d**) XPS high resolution scan of C1, (**e**) N1 and (**f**) full survey spectrum shows three peaks of carbon, nitrogen and oxygen.

Fourier-transform infrared spectroscopy was used to characterize the functional groups present on the nanosheets. The FTIR spectra (Figure 2a) shows a series of broad peaks at 3400 and 3000 cm^−1^ both characteristics of the stretching vibration of the N–H bond [38]. The vibrations imply that residual -NH or -NH_2_ groups remain in the as-obtained g-CN nanosheets [39]. Peaks at 1232, 1312, 1433, and 1404 cm^−1^ correspond to the typical stretching vibration modes of C=N, while bands near 1544 and 1632 cm^−1^ are ascribed to C–N stretching [40]. The sharp peak at 807 cm^−1^ is an out of plane bending vibration characteristic of the tri-s-triazine unit [37,38,39]. The results indicate that amino functionality is still present, and the surface functional groups of g-CN were retained after exfoliation. The TEM and AFM observations corroborate the FTIR results.

The phase composition of g-CN is revealed by the XRD pattern (Figure 2b). Two peaks occur at 2θ = 13.27° and 27.25° which correspond to the structural integrity of the packing motif of the tris-triazine units (100) and the conjugated aromatic system (002), respectively [25]. The SEM, TEM, and AFM images also support this observation. These data is firm evidence that few-layer g-CN nanosheets were successfully achieved. The XRD results are also in agreement with results reported elsewhere [27,30,36]. The graphitic nature of the material is also revealed in the Raman spectra (Figure 2c), where the two diagnostic peaks at 1365 and 1547 cm^−1^ correspond to disordered amorphous carbon (D band) and graphite (G band), respectively [41]. The band at 1547 cm^−1^ corresponds to the C=N stretching vibration and explains the graphitic G band and reveals the graphitic nature of the sample (Figure 2c). The above results confirm the intactness of the carbon backbone of the as-synthesized material with peculiar features and signature peaks.

The XPS core level spectra of the g-CN nanosheets show that the structure of carbon and nitrogen atoms in the as-synthesized sample are similar to the g-CN structure. In the high-resolution XPS spectra (Figure 2d,e) two distinct peaks are located near 284.86 and 288.15 eV in the C1s spectrum. The peak at 284.86, is typical for sp^2^ C–C bonds, while the peak at 288.15 eV corresponds to a sp^2^-bond carbon in a N-containing aromatic ring (N=C-N), i.e., the backbone of the carbon species in the g-CN nanosheets [42]. The N1s spectrum can be deconvoluted into three peaks: 398.6 eV, 400.1 eV, and 401.2 eV. The main peak at 398.6 eV corresponds to sp^2^ bonded N atom in the triazine rings (N=C-N), while the lower-intensity peak at 400.1 eV is either a tertiary nitrogen N atom in N–(C)_3_ or a N atom bonded to an H atom. The peak at 401.2 eV suggests the presence of an amino-functional group (C–N–H), originating from the defective condensation of the melon structures. It has been reported that tri-s-triazine units are interconnected with amino groups between the sheets [42]. In the survey spectrum (Figure 2f), only C, N, and O are observed. The O1s signal is likely atmospheric H_2_O, or CO_2_ molecules adsorbed on the g-CN surface—something that is confirmed by FTIR (Figure 1a) [43].

### 3.2. Effect of pH on the Binding of Hg^2+^ with g-CN

**Figure 3:**
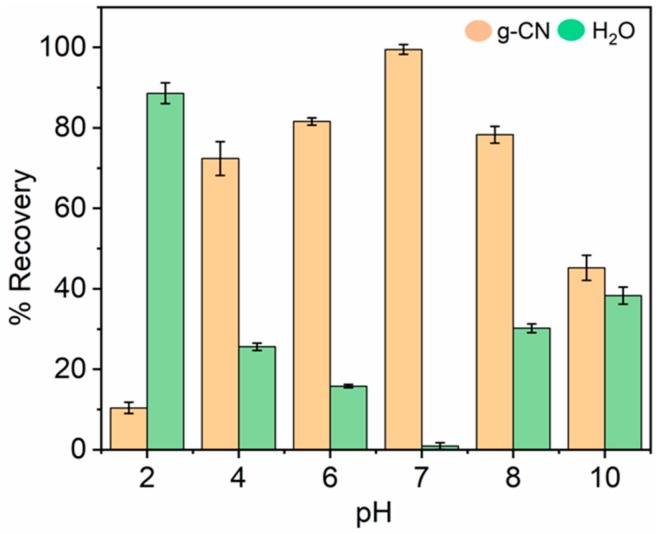
The % recovery vs pH (2–10) under identical conditions.

It is known from the literature that pH is a key factor influencing the binding of Hg^2+^ at the water adsorbent interface [44]. The adsorption mechanism in the colloidal liquid interaction process depends on the nature of the adsorbent surface, the target analyte and competition between target ions and the hydrogen bonds on the available active binding sites on the adsorbent surface [45,46]. This study investigated the adsorption of Hg^2+^ over a range of pH values from pH 2 to 10. Figure 3 shows the effect of pH on the binding efficiency of Hg^2+^ at a concentration of 100 ng mL^−1^. The results show how adsorption increases slowly from pH 2.0 to 6.0, with a sharp increase from pH 6.0 to 7.0. A similar trend was observed by [47] and [48]. The highest adsorption efficiency was observed at pH 7. In an acidic solution, the concentration of H^+^ ions increases. The effect is an increase in the competition between Hg^2+^ and H^+^ ions for the active sites and as a result adsorption efficiency decreases (Figure 3). Contrary to this, at pH 7 and above (pH 8 and pH 10), the H^+^ ions can be consummated and permit the reaction to occur; however, Hg^2+^ ions in the solution precipitate as the colloidal precipitate Hg(OH)_2_. Also, the formation of insoluble metal hydroxide species instead of free Hg^2+^ ions decreases adsorption efficiency. In this case, the experiment did not include pH values above pH 10, and the increasing binding efficiency with increasing pH can be explained by a decrease in competition between H^+^ and the positively charged Hg^2+^ on the g-CN surface. At low pH (pH < 2–4), excessive protonation of nitrogen’s lone pair of electrons occurs, resulting in a decrease in the number of available sorption sites for Hg^2+^ [48]. A pH of 7 is optimal for Hg^2+^ adsorption.

### 3.3. Effect of Contact Time

**Figure 4:**
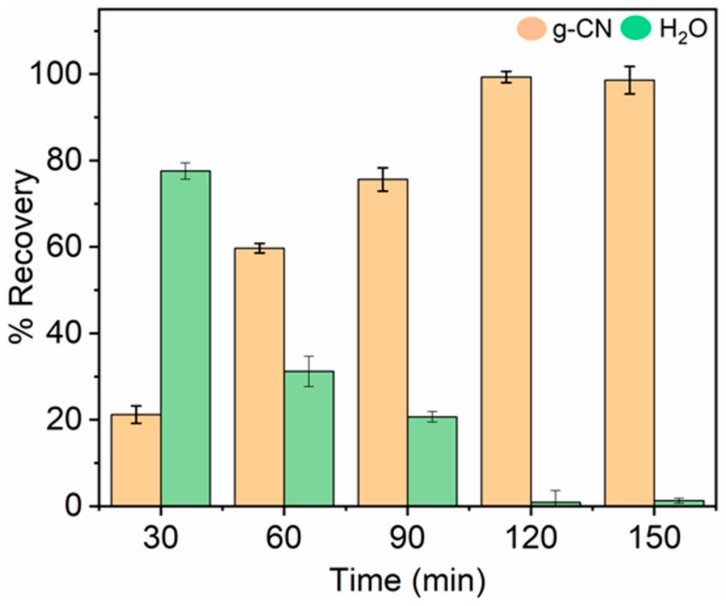
Effect of contact time on the binding of Hg^2+^ (100 ng mL^−1^) under ideal conditions. Agitation speed = 210 (rpm), room temperature = 21 °C and optimized pH-7.

To establish the optimal binding time of Hg^2+^ and to understand better the adsorption process, adsorption was studied as a function of contact time (Figure 4). The adsorption patterns describe the binding rate of the Hg^2+^ on the g-CN and is an important characteristic for defining adsorption efficiency and helps in understanding better the adsorption mechanism [49,50,51]. Figure 4 shows the adsorption profiles of Hg^2+^ vs time. The adsorption on g-CN nanosheets occurs slowly at first (<60 min) and then increases with 99% recovery being achieved at around 120 min. After 120 min, time no longer has an effect on adsorption since the binding sites on the g-CN nanosheets are saturated. This phenomenon is commonly observed in adsorption studies [52].

### 3.4. Effect of g-CN Concentration on Hg^2+^ Adsorption

**Figure 5:**
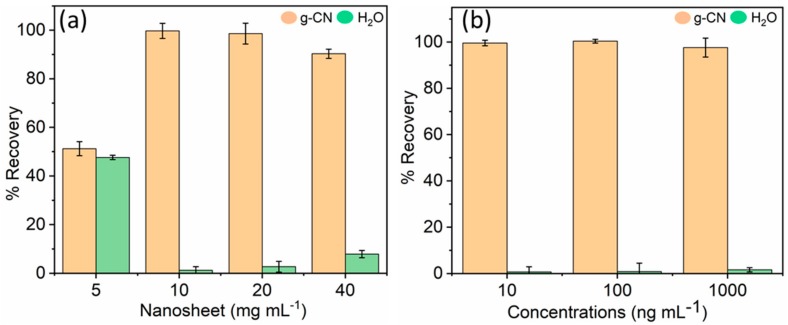
Optimal studies of g-CN nanosheets at (**a**) different concentration of g-CN (5, 10, 20, and 40 mg mL^−1^) required to saturate Hg^2+^ (100 ng mL^−1^) under optimised incubation and (**b**) binding efficiency of g-CN nanosheets (10 mg mL^−1^) at different concentration of Hg^2+^ (pH 7). The sample volume was 3 mL.

The amount of g-CN is also an important parameter in the determination of binding percentage and the binding efficiency of Hg^2+^. Figure 5a shows the amount of g-CN vs the amount of Hg^2+^ adsorbed by the nanosheets. The results suggest that at an optimised concentration (100 ng mL^−1^) of Hg^2+^, the binding per cent increases as the g-CN content increases. This increase can be attributed to an increase in surface area, greater competition for binding sites at a higher dose or an insufficient amount of Hg^2+^ in solution compared to the number of available binding sites. The decrease in binding efficiency (5 mg mL^−1^) is because some of the adsorption sites remain unsaturated during the adsorption process (Figure 5a). The maximum amount of Hg^2+^ adsorbed was attained at about 10 mg mL^−1^ of g-CN and remained the same, even after increasing the amount of g-CN to 20 mg mL^−1^ (Figure 5a). Any further increase in the g-CN (40 mg mL^−1^) has a stalking or aggregation effect in the solution, which affects the adsorption of Hg^2+^. Using an optimal concentration of g-CN (10 mg mL^−1^), different amounts of Hg^2+^ were tested (Figure 5b). The synthesised nanosheet effectively adsorbs all three concentration (10, 100 and 1000 ng mL^−1^) of Hg^2+^ (Figure 5b). It is worth noting that the amount of g-CN nanosheets used in this study are much lower than for most of the nanomaterials based adsorbents used for adsorbing of Hg^2+^ ions in water, while the adsorption efficiency of the g-CN obtained by the modified polycondensation process is comparable with that of other adsorbent materials reported in the literature [16,46,53,54,55,56,57,58,59,60,61,62,63,64,65,66].

### 3.5. Determination of Hg^2+^ in Different Matrices

**Figure 6:**
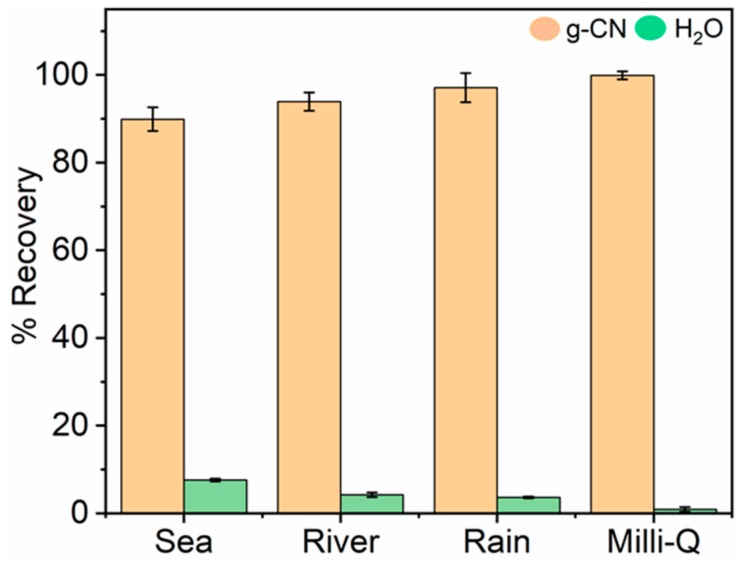
Adsorption studies using sea, river, rain, and Milli-Q water spiked with 100 ng mL^−1^ of Hg^2+^. The sample volume was 3 mL.

Adsorption experiments were performed in samples of sea, river, rain, and Milli-Q quality water to test the practical application of the g-CN nanosheets for adsorbing Hg^2+^ (Figure 6). The procedure and handling of samples were performed using prescribed standard protocols [33]. The samples were spiked with a known concentration of Hg^2+^ (100 ng mL^−1^) solution. Good recoveries were achieved from each of the four matrices: 89%, 93%, 97%, and 100%, respectively (Figure 6). The lower recovery (89%) from seawater can be explained by the higher salinity and presence of solid particles that can interfere with recovery by competing with Hg^2+^ for the binding sites on g-CN surfaces (Figure 6). Compared to seawater, river (93%) and rainwater (97%) have relatively better recoveries. The protonation of ions in the solution is also an essential parameter in determining binding efficiency. [67] Approximately, 100% the Hg^2+^ was removed from the Milli-Q quality water.

**Figure 7:**
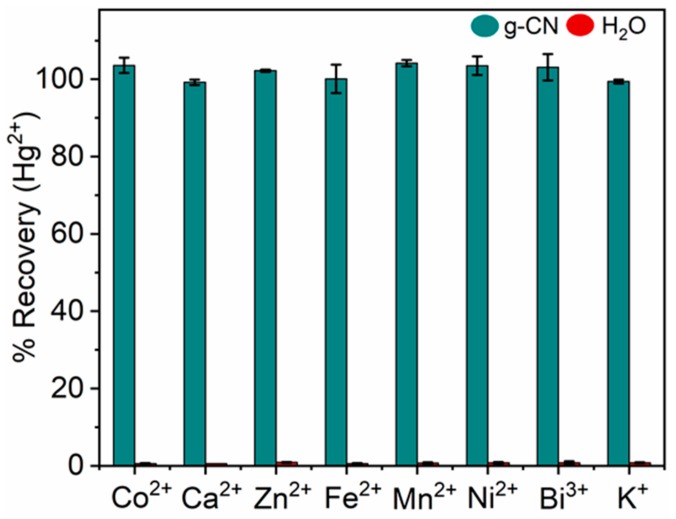
Potential influence of metal ions on the % recovery of Hg^2+^ (100 ng mL^−1^ Hg^2+^ and 500 ng mL^−1^ of Co^2+^, Ca^2+^, Zn^2+^, Fe^2+^, Mn^2+^, Ni^2+^, Bi^3+^, Na^+^, and K^+^).

Selective detection of Hg^2+^ is challenging due to the presence of different metal ions in environmental samples. Therefore, the selectivity toward the Hg^2+^ ions was determined by measuring recovery in the presence of nine possible interfering ions: Co^2+^, Ca^2+^, Zn^2+^, Fe^2+^, Mn^2+^, Ni^2+^, Bi^3+^, Na^+^, and K^+^. Figure 7 shows the % recovery of Hg^2+^ in the presence of each metal ion. The concentration for each interfering ion was 500 ng mL^−1^ and 100 ng mL^−1^ for Hg^2+^. The results (Figure 7) showed little or no interference by any of the interfering metal ions and that the g-CN sheets are highly selective towards Hg^2+^ under optimum conditions. This selectivity originates from the coordination between the –NH/NH_2_ and the Hg^2+^ ions [68].

### 3.6. Regeneration of g-CN for Repeated Trials

**Figure 8:**
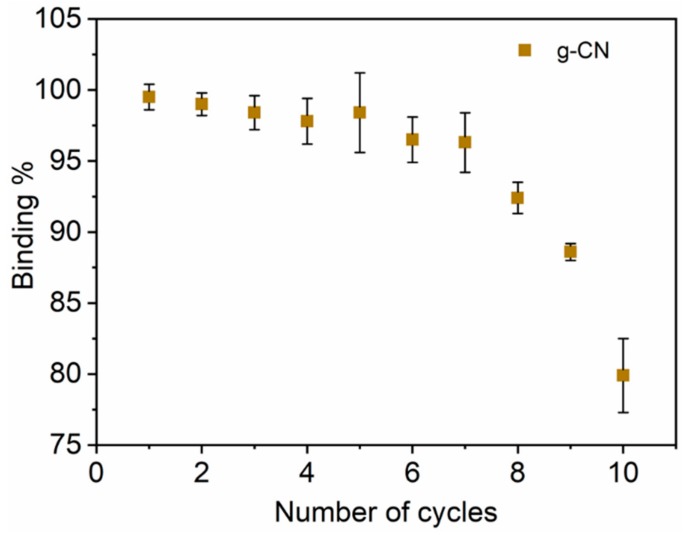
Adsorption–desorption regeneration cycling of g-CN.

The ability to regenerate the g-CN nanosheets using sorption and desorption cycles for Hg^2+^ is essential for their long-term use, eco-optimisation, and for reducing device costs. Stable g-CN sheets also open doors for other opportunities, for example, in sensor applications as potential transducers. In this case, the adsorbent was regenerated using HCl (0.5 M), and 90% ethanol. Figure 8 shows the binding efficiency vs the number of regeneration cycles measured in pure water. The sorption efficiency of g-CN decreases by about 7.3% over seven cycles, after which there is a significant drop-off although even after ten cycles the amount of Hg^2+^ adsorbed is only reduced by 20%. This reduction is likely due to the reduce binding efficiency of the nanosheet by repeated washing and regenerating steps. The overall results indicate that g-CN is chemically stable and can be regenerated, which makes it a promising candidate for reusable PAS (adsorbent) and sensor (transducer) applications.

## 4. Conclusions

Simple, cost-effective, and greener interweaved nano-knitted g-CN nanosheets were developed for the adsorption of Hg^2+^ from different aqueous matrices. A Modified method utilizing fast polycondensation and rapid exfoliation produced nanosheets with a > 70% yield. The high content of reactive functional species selectively bound Hg^2+^ without the need for spacer ligands, which offers many advantages over existing Hg^2+^ passive sampling adsorbents and transducers in Hg sensors. The g-CN nanosheets were able to rapidly bind and remove Hg^2+^ ions from aqueous solutions at environmental pH’s due to the high surface area, porosity, and internal diffusion resistance. The g-CN nanosheets also have higher adsorption efficiencies and shorter reaction times compared to other available adsorbents. It was also possible to recover the material (adsorption efficiency 99.5%) using HCl (0.5 M), and 90% ethanol. The adsorbent can also be regenerated up to 10 times. The present approach presented here is economical and straightforward, and no further chemical modification or additional oxidant is required, which simplifies the operation process. The reliability of the g-CN was ascertained using real aqueous sample matrices (sea, river and Milli-Q quality water) to confirm its applicability for environmental monitoring applications. Moreover, g-CN nanosheets offer many benefits including scalability, robustness, rapidity, sensitivity, and selectivity, making this material potentially useful for environmental and clinical sensors and diagnosis by using g-CN based hybrid films as biocatalysts.
